# Resuscitation discussion practices: a survey of European geriatricians

**DOI:** 10.1007/s41999-025-01218-8

**Published:** 2025-05-05

**Authors:** Johannes Trabert, Caterina Trevisan, Nienke Golüke, Guillaume Chapelet, Elisabet Sanchez-Garcia, Mary Ni Lochlainn

**Affiliations:** 1 Department of Geriatric Medicine, AGAPLESION Markus Hospital, Frankfurt, Germany; 2 Department of Medical Sciences, University of Ferrara, Ferrara, Italy; 3 Department of Geriatric Medicine, Hospital Gelderse Vallei, Ede, The Netherlands; 4 Clinical Gerontology Department, CHU Nantes, Nantes, France; 5 Mater Private Network, Cork, Ireland; 6 Hospital Ramon y Cajal, Madrid, Spain; 7 Centre for Ageing Resilience in a Changing Environment, Department of Twin Research and Genetic Epidemiology, King’s College London, London, UK

**Keywords:** Cardiopulmonary resuscitation, CPR, DNAR, DNR, Communication, Geriatrics

## Abstract

**Purpose:**

Cardiopulmonary resuscitation (CPR) is a life-saving treatment, yet outcomes in frail older adults are poor. Despite this, decision-making around resuscitation remains complex, influenced by misperceptions among patients, families, and physicians. We aimed to describe current practices, identify difficulties and barriers regarding do-not-resuscitate (DNR) decisions, and to assess differences across Europe.

**Methods:**

A cross-sectional online survey was conducted between September and December 2023, targeting geriatricians across Europe. The survey, disseminated via professional networks, assessed frequency and confidence in discussing DNR orders, barriers to discussions, and inter-country differences. Sociodemographic data and responses were analysed using descriptive statistics and logistic regression to identify factors influencing resuscitation discussions.

**Results:**

A total of 473 geriatricians participated across 22 countries (65% female, mean age 41.8 years, mean experience 10.5 years). Discussions of DNR orders occurred with ≥ 60% of hospitalised patients for 40% of respondents, with confidence discussing DNR orders reported by 80%. Reported barriers included time constraints (50%), cultural beliefs (25%), and lack of training (41%). Patients were kept ‘for resuscitation’ despite medical futility in an estimated 38% of cases at the request of patients or next of kin. Western European geriatricians discussed DNR orders more frequently and confidently than peers in other regions. Experience (> 20 years) was strongly associated with confidence (OR 7.53, 95% CI 1.93–32.35).

**Conclusion:**

Significant variability exists in resuscitation discussions among European geriatricians, influenced by region, gender, and experience. Enhanced physician training, societal awareness, and standardised protocols could improve resuscitation decision-making and reduce inappropriate resuscitation attempts.

**Supplementary Information:**

The online version contains supplementary material available at 10.1007/s41999-025-01218-8.

## Key summary points

**Aim:**

To investigate describe current practices, identify difficulties and barriers regarding DNR decisions, and to assess differences across Europe.

**Findings:**

European DNR decision-making reveals stark regional differences, with Western European and highly experienced geriatricians showing greater confidence and frequency in discussions, though most faced barriers like time constraints and lack of training.

**Message:**

Standardised protocols, enhanced physician training, and targeted public awareness campaigns are essential for optimizing DNR decision-making.

**Supplementary Information:**

The online version contains supplementary material available at 10.1007/s41999-025-01218-8.

## Background

Cardiopulmonary Resuscitation (CPR) is performed as an acute life-saving treatment every day, across the world. Survival to hospital discharge after CPR in older adults ranges from 11.6 to 28.5% for in-hospital arrests and 0–11.1% for out-of-hospital arrests [1], with rates declining with age [1, 2].

One of the key influencing factors on survival is existing frailty, with non-frail older people showing much higher rates of survival compared to people living with frailty or severe frailty [3, 4]. In contrast to frailty, age does not seem to be as good a discriminator in CPR outcomes [5]. While one meta-analysis did report age as a negative prognostic factor [6], others have found no significant association between survival post-CPR and age [7].

In clinical practice, there is usually a triad involved in the decision making around resuscitation, the patient, their next of kin/family members and the treating medical team. The perceptions of all three regarding resuscitation will influence discussions, and subsequent decisions. An American study found that patients significantly reduced their desire for CPR after learning of its low success rate, with patients aged 86 years or older having the lowest CPR preference [8].

Discrepancies between patient and next-of-kin wishes regarding CPR are common In a systematic review, 16 studies involving 151 hypothetical scenarios and 2595 surrogate-patient pairs found disagreements in one third of cases [9]. This highlights the need for open discussions about CPR outcomes and end-of-life care preferences between patients, their families, and medical professionals. The communication skills of the treating medical team are of huge value in these discussions, with potential for emotional distress among patients and/or their next of kin if not communicated clearly and sensitively.

There is also evidence of misperceptions regarding the outcome of CPR among physicians. A sub-analysis of an international multicentre survey investigating 611 cases of out-of-hospital CPR in patients aged 80 years or older demonstrated that 45% of physicians considered CPR appropriate even if cardiac arrest was unwitnessed and unshockable. The survival of these patients was 0%. Only 25% considered CPR inappropriate in this case [10]. Clearly, a mismatch can exist between the perceptions of patients, their next-of kin, and within the medical teams, in relation to resuscitation, and the utility, or futility, of CPR.

Implementation of structured decision-making, with accompanying timely and structured documentation may be a way to resolve this mismatch [11, 12]. Methods to improve the quality of resuscitation decisions have included training of physicians in communication skills, training of physicians and nursing home staff regarding advance care planning, introduction of standardised do-not-resuscitate (DNR) protocols and their structured discussions with patients and DNR preferences being established at the point of hospital admission [13]. Even though physicians often feel a DNR decision should be made, proper documentation is often delayed or lacking, potentially leading to unwanted and/or futile resuscitation attempts [14].

A wide gap in the implementation of resuscitation decision-making has been shown across European countries. For example, in Switzerland 73% of deceased patients had DNR decisions available, while in Italy this was the case in only 16% [15]. As geriatricians treat older and frail patients, advance care planning and decision-making around resuscitation is of the utmost importance to this professional group. To our knowledge, there has been no research on current practice regarding resuscitation decision-making among geriatricians in Europe. In this study, we aimed to describe current resuscitation practices among geriatricians across Europe, to identify difficulties and barriers regarding DNR decisions for physicians, and to assess inter-country differences across the continent, to facilitate country-specific learning.

## Methods

Between September 1 st and December 31 st, 2023, an online survey of geriatricians across Europe was carried out. The survey was designed by a team of geriatricians representing a range of European countries (Germany, France, The Netherlands, Spain, United Kingdom, Ireland, and Italy) and included questions aimed at investigating the current practice concerning resuscitation decision-making among European physicians working in geriatric medicine. The survey was developed by the research team through internal discussion until consensus was reached. Particular attention was paid to possible cultural differences between European countries, and the wording of questions was chosen with the consideration that English would not be the first language of many respondents in mind.

The survey was administered in English through a Google form, with anonymous data collection on a voluntary basis. Survey dissemination was performed through social media and newsletters of the main European geriatric societies (European Geriatric Medicine Society, British Geriatric Society, Irish Gerontological Society, Italian Society of Gerontology and Geriatrics, German Geriatric Society, Dutch Geriatric Society, Spanish Society of Geriatrics and Gerontology and the French Society of Geriatrics and Gerontology) from September 1 st to December 31 st, 2023. Dissemination was also carried out using the European Academy of Medicine in Ageing (EAMA) network, as all authors are alumni. The study was approved by the ethics review board of the State Hesse, Germany (2023-3375-AF). All participants gave informed consent to take part in the survey.

### Data collection

**Fig. 1:**
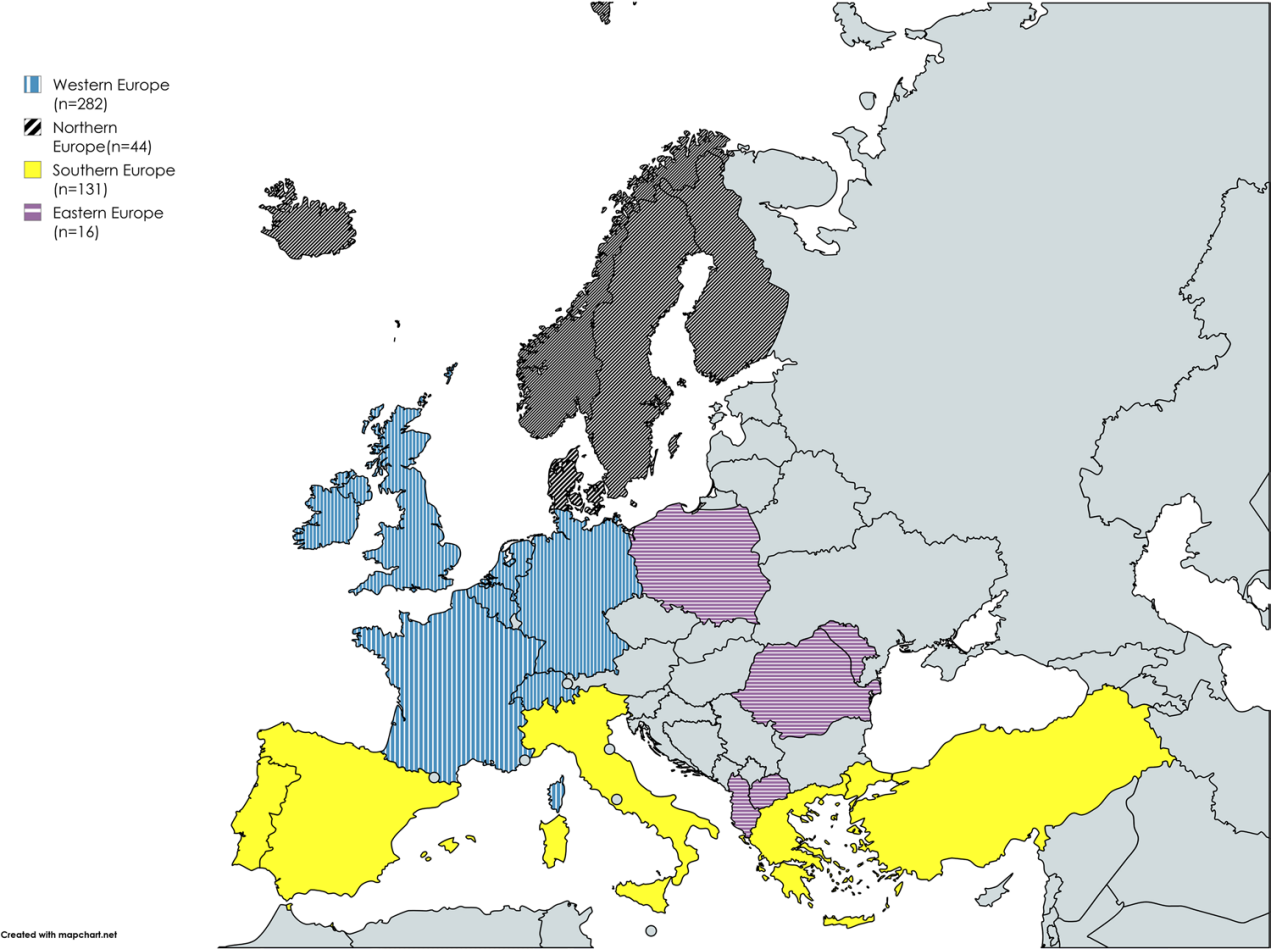
European map with the number of participants per region. Eastern Europe (*n* = 16): Albania, Moldova, North Macedonia, Poland, Romania. Western Europe (*n* = 282): Belgium, France, Germany, Ireland, The Netherlands, Switzerland, the United Kingdom. Northern Europe (*n* = 44): Denmark, Finland, Iceland, Norway, Sweden. Southern Europe (*n* = 131): Italy, Spain, Portugal, Greece, Turkey

From each participant, we collected sociodemographic information, including age, sex, working experience as a medical doctor and as a geriatrician, and country of practice. Countries were grouped into Northern (Denmark, Finland, Iceland, Norway and Sweden), Eastern (Albania, Moldova, North Macedonia, Poland, and Romania), Western (Belgium, France, Germany, Ireland, The Netherlands, Switzerland, and the United Kingdom), and Southern Europe (Italy, Spain, Portugal, Greece, and Turkey), see Fig. 1. The questionnaire included 16 questions investigating the frequency and confidence in discussing DNR orders in patients admitted to hospital, training received in resuscitation discussions, perceived barriers in discussing DNR orders and possible solutions for improving these discussions. The administered survey questions can be seen in Table 2.

### Statistical analysis

The characteristics of the survey participants and their responses were expressed as mean and standard deviation (SD) for quantitative variables, and as number and frequencies (%) for categorical variables. Comparison of responses between individuals working in different European countries was performed through Chi-square test or ANOVA, for categorical and quantitative variables, respectively. The assessment of the factors associated with a high frequency (considering ≥ 60% of hospitalised patients as a cutoff in order to identify those who discussed DNR orders with the majority of patients) and confidence (strongly agree or agree with the statement “I feel confident discussing DNR orders with patients”) in discussing DNR orders was performed through binary logistic regression. Independent variables included age, sex, geographical area of residence in Europe, and years of experience as a geriatrician. In sensitivity analyses, we changed the reference group based on geographical area in order to compare different European countries with one other. The strength of these associations was expressed as Odds Ratio (OR) and 95% Confidence Interval (95% CI). All statistical analyses were two-tailed and a p-value < 0.05 was considered statistically significant. Analyses were conducted using the R statistical software - version 4.3.3 [16].

## Results

The survey was completed by 473 respondents, with a mean age of 41.8 years, across 22 countries. Of the survey respondents, 282 were working in Western Europe (59.6% of the sample), 16 in Eastern Europe (3.4%), 44 in Northern Europe (9.3%), and 131 (27.7% of the sample) in Southern European countries (see Fig. 1). Of these, 65% were female and the respondents had a mean of 10.5 years of experience in geriatric medicine. Baseline characteristics of respondents are presented in Table 1.

**Table 1:** Baseline characteristics of respondents

Baseline characteristics	Frequency n = 473
Age in years, mean (± SD)	41.8 (± 9.86)
Female, n (%)	305 (64.9)
Years of experience in geriatric field, mean (± SD)	10.5 (± 8.2)
Years of experience in geriatric field, n (%)	
< 10	281 (59.4)
10–19	108 (22.8)
≥ 20	84 (17.8)
Countries, n (%)	
Albania	1 (0.2)
Belgium	11 (2.3)
Denmark	17 (3.6)
Finland	11 (2.3)
France	13 (2.7)
Germany	83 (17.5)
Greece	4 (0.8)
Iceland	1 (0.2)
Ireland	45 (9.5)
Italy	56 (11.8)
Moldova	2 (0.4)
The Netherlands	32 (6.8)
North Macedonia	2 (0.4)
Norway	12 (2.5)
Poland	7 (1.5)
Portugal	6 (1.3)
Romania	4 (0.8)
Spain	60 (12.7)
Sweden	3 (0.6)
Switzerland	20 (4.2)
Turkey	5 (1.1)
United Kingdom	78 (16.5)

## Survey results

The survey results are summarised in Table 2, by European area. Full survey results are presented in Supplementary Table 1, and by individual country in Supplementary Table 2. 23% of the sample reported discussing resuscitation in the majority (80–100%) of hospitalised patients, while 20% discussed it in a minority (< 20%) of patients. Of the respondents, 55% reported having structured documentation and standardised protocols regarding Do Not Resuscitate (DNR) orders. Almost half of the respondents (48%) of the sample stated that the most appropriate time to discuss resuscitation was upon admission. 59% reported preferences of resuscitation were not routinely recorded at hospital admission.

**Table 2:** Respondents’ characteristics and survey results by European area

European area	Western	Eastern	Northern	Southern	p
n	282	16	44	131	
Age, mean (SD)	41.9 (9.4)	41.4 (12.3)	44.1 (8.0)	41.1 (11.1)	0.387
Female sex, n (%)	175 (62.1)	13 (81.2)	27 (61.4)	90 (70.3)	0.19
Years of experience in geriatric field, mean (SD)	10.2 (8.0)	11.75 (9.1)	10.36 (6.7)	11.1 (10.0)	0.68
With which proportion of admitted patients, independently of morbidity status, do you discuss DNR orders?					< 0.001
0–20%	26 (9.2)	11 (68.8)	5 (11.6)	54 (41.2)	
20–40%	52 (18.4)	0 (0.0)	12 (27.9)	26 (19.8)	
40–60%	60 (21.3)	2 (12.5)	12 (27.9)	24 (18.3)	
60–80%	55 (19.5)	2 (12.5)	4 (9.3)	18 (13.7)	
80–100%	89 (31.6)	1 (6.2)	10 (23.3)	9 (6.9)	
I feel confident discussing DNR orders with patients					< 0.001
Strongly agree	130 (46.1)	0 (0.0)	17 (38.6)	14 (10.7)	
Agree	135 (47.9)	5 (31.2)	22 (50.0)	54 (41.2)	
Neutral	11 (3.9)	4 (25.0)	4 (9.1)	28 (21.4)	
Disagree	6 (2.1)	5 (31.2)	1 (2.3)	30 (22.9)	
Strongly disagree	0 (0.0)	2 (12.5)	0 (0.0)	5 (3.8)	
I feel confident discussing DNR orders with patient’s next of kin					< 0.001
Strongly agree	125 (44.3)	0 (0.0)	14 (31.8)	41 (31.3)	
Agree	137 (48.6)	11 (68.8)	26 (59.1)	60 (45.8)	
Neutral	18 (6.4)	2 (12.5)	4 (9.1)	20 (15.3)	
Disagree	2 (0.7)	2 (12.5)	0 (0.0)	10 (7.6)	
Strongly disagree	0 (0.0)	1 (6.2)	0 (0.0)	0 (0.0)	
What do you feel are the barriers or reasons not to discuss resuscitation or talking about death and dying?					
Religious or cultural beliefs	68 (24.1)	6 (37.5)	8 (18.2)	35 (26.7)	0.432
Fear of lawsuits	12 (4.3)	4 (25.0)	1 (2.3)	27 (20.6)	< 0.001
Inexperience or lack of skills	12 (4.3)	5 (31.2)	1 (2.3)	42 (32.1)	< 0.001
Embarrassment/feeling uncomfortable	41 (14.5)	5 (31.2)	8 (18.2)	45 (34.4)	< 0.001
Lack of time	160 (56.7)	6 (37.5)	32 (72.7)	38 (29.0)	< 0.001
I don’t believe it’s needed	32 (11.3)	2 (12.5)	7 (15.9)	3 (2.3)	0.009
Other	46 (16.3)	3 (18.8)	11 (25.0)	11 (8.4)	0.037
When do you think discussing DNR orders in the hospital is most appropriate (for those patients without known ceilings of care)?					< 0.001
On admission	165 (58.5)	8 (50.0)	13 (29.5)	41 (31.3)	
A few days after admission	52 (18.4)	1 (6.2)	23 (52.3)	41 (31.3)	
In case of clinical deterioration	30 (10.6)	7 (43.8)	2 (4.5)	43 (32.8)	
Other	35 (12.4)	0 (0.0)	6 (13.6)	6 (4.6)	
With whom do you usually discuss DNR orders for patients with full capacity to make a decision?					< 0.001
Patients	162 (57.4)	1 (6.2)	28 (63.6)	27 (20.6)	
Next of kin	1 (0.4)	7 (43.8)	0 (0.0)	34 (26.0)	
Both patients and next of kin	118 (41.9)	5 (31.2)	15 (34.1)	65 (49.6)	
Neither	0 (0.0)	3 (18.8)	1 (2.3)	4 (3.1)	
Missing	1 (0.4)	0 (0.0)	0 (0.0)	1 (0.8)	
With whom do you usually discuss DNR orders for patients with impaired capacity to make a decision?					< 0.001
Patients	13 (4.6)	1 (6.2)	2 (4.5)	3 (2.3)	
Next of kin/legal representative	77 (27.3)	8 (50.0)	10 (22.7)	86 (65.6)	
Both patients and next of kin	191 (67.7)	4 (25.0)	30 (68.2)	37 (28.2)	
Neither	0 (0.0)	3 (18.8)	2 (4.5)	2 (1.5)	
Missing	1 (0.4)	0 (0.0)	0 (0.0)	3 (2.3)	
Is it legal in your country to make DNR decisions by medical opinion alone (e.g., patient has cognitive impairment, lacks capacity, and no legal representative available)?					< 0.001
Yes	228 (80.9)	6 (37.5)	44 (100.0)	63 (48.1)	
No	24 (8.5)	7 (43.8)	0 (0.0)	29 (22.1)	
I don’t know	30 (10.6)	3 (18.8)	0 (0.0)	38 (29.0)	
Missing	0 (0.0)	0 (0.0)	0 (0.0)	1 (0.8)	
I feel confident making DNR decisions based on medical opinion alone (e.g. in the example above)?					< 0.001
Strongly agree	70 (24.8)	1 (6.2)	12 (27.3)	7 (5.3)	
Agree	131 (46.5)	6 (37.5)	28 (63.6)	59 (45.0)	
Neutral	38 (13.5)	5 (31.2)	4 (9.1)	26 (19.8)	
Disagree	37 (13.1)	4 (25.0)	0 (0.0)	33 (25.2)	
Strongly disagree	5 (1.8)	0 (0.0)	0 (0.0)	5 (3.8)	
Missing	1 (0.4)	0 (0.0)	0 (0.0)	1 (0.8)	
If there is an advance care plan mentioning a conditional DNR in specific situations, how often do you find it difficult to know whether it should apply in the patient’s current situation?					0.186
Always	11 (3.9)	1 (6.2)	0 (0.0)	5 (3.8)	
Often	90 (31.9)	5 (31.2)	13 (29.5)	61 (46.6)	
Rarely	131 (46.5)	5 (31.2)	22 (50.0)	52 (39.7)	
Never	12 (4.3)	2 (12.5)	4 (9.1)	4 (3.1)	
Not applicable	35 (12.4)	3 (18.8)	5 (11.4)	8 (6.1)	
Missing	3 (1.1)	0 (0.0)	0 (0.0)	1 (0.8)	
In a situation where the medical team thinks that a therapy is futile (e.g. resuscitation), how often would this therapy anyway be performed in your country, if the patient or next of kin mandates it?					< 0.001
Always	7 (2.5)	2 (12.5)	0 (0.0)	12 (9.2)	
Often	88 (31.2)	7 (43.8)	5 (11.4)	58 (44.3)	
Rarely	167 (59.2)	6 (37.5)	34 (77.3)	57 (43.5)	
Never	12 (4.3)	1 (6.2)	5 (11.4)	2 (1.5)	
Not applicable	6 (2.1)	0 (0.0)	0 (0.0)	1 (0.8)	
Missing	2 (0.7)	0 (0.0)	0 (0.0)	1 (0.8)	
How often do you re-evaluate DNR orders after making the decision initially (during the same hospitalisation)?					< 0.001
Always	8 (2.8)	1 (6.2)	1 (2.3)	6 (4.6)	
Often	78 (27.7)	5 (31.2)	8 (18.2)	54 (41.2)	
Rarely	190 (67.4)	6 (37.5)	30 (68.2)	65 (49.6)	
Never	6 (2.1)	4 (25.0)	4 (9.1)	5 (3.8)	
Missing	0 (0.0)	0 (0.0)	1 (2.3)	1 (0.8)	
Is it mandatory in your country to write and assess preferences regarding resuscitation in clinical records on admission?					0.006
Yes	92 (32.6)	1 (6.2)	14 (31.8)	25 (19.1)	
No	156 (55.3)	12 (75.0)	27 (61.4)	83 (63.4)	
I don’t know	34 (12.1)	2 (12.5)	2 (4.5)	20 (15.3)	
Missing	0 (0.0)	1 (6.2)	1 (2.3)	3 (2.3)	
Does your hospital provide a standardised DNR document?					< 0.001
Yes	225 (79.8)	4 (25.0)	21 (47.7)	10 (7.6)	
No	48 (17.0)	12 (75.0)	21 (47.7)	109 (83.2)	
I don’t know	8 (2.8)	0 (0.0)	2 (4.5)	11 (8.4)	
Missing	1 (0.4)	0 (0.0)	0 (0.0)	1 (0.8)	
Discussing DNR orders in my country is well accepted					< 0.001
Strongly agree	64 (22.7)	0 (0.0)	14 (31.8)	7 (5.3)	
Agree	151 (53.5)	1 (6.2)	21 (47.7)	33 (25.2)	
Neutral	49 (17.4)	6 (37.5)	7 (15.9)	29 (22.1)	
Disagree	17 (6.0)	5 (31.2)	2 (4.5)	50 (38.2)	
Strongly disagree	0 (0.0)	4 (25.0)	0 (0.0)	10 (7.6)	
Missing	1 (0.4)	0 (0.0)	0 (0.0)	2 (1.5)	
Did you receive any training about communication/discussing DNR orders?					0.003
In university	15 (5.3)	3 (18.8)	3 (6.8)	6 (4.6)	
In residency/specialty training	107 (37.9)	4 (25.0)	21 (47.7)	38 (29.0)	
Both in university and residency/specialty training	60 (21.3)	2 (12.5)	8 (18.2)	13 (9.9)	
No training	100 (35.5)	7 (43.8)	12 (27.3)	73 (55.7)	
Missing	0 (0.0)	0 (0.0)	0 (0.0)	1 (0.8)	
In your opinion, what needs to change to improve DNR decision making in your country? (more than one answer possible)					
More training	180 (64.3)	11 (68.8)	30 (68.2)	107 (84.3)	0.001
Mandatory discussions	108 (38.3)	10 (62.5)	18 (40.9)	86 (66.2)	< 0.001
More awareness in society	240 (85.1)	14 (87.5)	36 (81.8)	97 (74.6)	0.073
Nothing	3 (1.1)	0 (0.0)	2 (4.5)	0 (0.0)	0.083
Other	13 (5.2)	1 (6.2)	2 (4.6)	9 (7.2)	0.698

Almost 80% of respondents agreed or strongly agreed to feeling confident in discussing resuscitation with patients and/or next of kin. In patients with capacity to make a decision, resuscitation discussions were held with patients in 46% of cases, and in 43%, the discussions were held with both the patients and their next of kin. For those with impaired capacity, resuscitation discussions were mostly held with both patients and next of kin (55%), or with next of kin or legal representative alone (39%). The most significant barrier to discussing resuscitation orders was identified as a lack of time (50%). Cultural beliefs were a barrier in 25% of cases, while 21% cited discomfort or lack of confidence as significant barriers.

Notably, in cases where the medical team deemed CPR futile, 38% of respondents indicated that the therapy would still be performed if requested by the next of kin or patient. Furthermore, 19% of respondents reported that discussing resuscitation was not well accepted in their country, which was especially pronounced in Southern and Eastern Europe. In terms of physician factors, 41% of respondents had no training in discussing resuscitation, while 70% felt more training might improve resuscitation decision making in their country. Other factors that might improve resuscitation decision making according to our respondents included more awareness in society (82%), and mandatory resuscitation discussions (47%).

## Frequency and confidence in discussing resuscitation

The results of the multivariable logistic regression analysis (see Table 3) revealed that factors associated with discussing resuscitation more frequently, in ≥ 60% of hospitalised patients, were female sex (OR 1.67, 95% CI 1.10–2.56, p = 0.018) and being from a Western European country in comparison with Northern (OR 0.43, 95% CI 0.21–0.85, p = 0.018) Eastern (OR 0.20, 95% CI 0.04–0.66, p = 0.016) and Southern European countries (OR 0.22, 95% CI 0.13–0.36, p < 0.001). The number of years working in geriatric medicine and age were not significantly associated with frequency of discussing resuscitation.

**Table 3:** Factors associated with frequency of discussing resuscitation and confidence in discussing resuscitation

	Univariate analyses odds radio (95% CI)	Multivariate analyses odds radio (95% CI)
Discussing resuscitation with ≥ 60% patients		
Age (years)	0.99 (0.98–1.01)	0.99 (0.96–1.03)
Female sex (vs male)	1.47 (1.00–2.19)	1.67 (1.10–2.56)*
European region		
Northern vs western	0.46 (0.23–0.90)*	0.43 (0.21–0.85)*
Eastern vs western	0.22 (0.05–0.70)*	0.20 (0.04–0.66)*
Southern vs western	0.25 (0.15–0.40)***	0.22 (0.13–0.36)***
Years of experience in geriatric field		
10–19 vs < 10	1.25 (0.79–1.95)	1.32 (0.76–2.30)
20 + vs < 10	0.71 (0.42–1.18)	1.03 (0.44–2.43)
Confidence in discussing resuscitation		
Age (years)	1.05 (1.02–1.08)*	1.01 (0.97–1.06)
Female sex (vs male)	1.00 (0.62–1.60)	2.03 (1.10–3.81)*
European area		
Northern vs western	0.50 (0.19–1.59)	0.55 (0.20–1.77)
Eastern vs western	0.03 (0.01–0.09)***	0.02 (0.00–0.06)***
Southern vs western	0.07 (0.04–0.12)***	0.05 (0.03–0.09)***
Work experience in geriatric medicine (years)		
10–19 vs < 10	1.22 (0.72–2.14)	0.85 (0.37–1.94)
20 + vs < 10	3.44 (1.61–8.53)**	7.53 (1.93–32.35)**

The multivariate analyses are adjusted for all other variables in this table

*p-value < 0.05, **p-value < 0.01, ***p-value < 0.001

Female physicians (OR 2.03, 95% CI 1.10–3.81, p = 0.024), and those with ≥ 20 years of experience in geriatric medicine (OR 7.53, 95% CI 1.93–32.35, p = 0.005) were more likely to report feeling confident in discussing resuscitation. Compared with those working in a Western European country, physicians in the Eastern (OR 0.02, 95% CI 0.00–0.06, p < 0.001) or Southern (OR 0.05, 95% CI 0.03–0.09, p < 0.001) countries were less likely to feel confident. On the other hand, no significant results were observed for the Northern countries or when considering the respondents’ age.

While western countries were used as the reference category here, Supplementary Tables 3 and 4 present the findings of the multivariable logistic regression analysis for factors associated with frequency of discussing resuscitation and factors associated with confidence in discussing resuscitation respectively, with each different European area as the reference category.

## Discussion

Resuscitation discussions, particularly among older adults and those living with frailty, represent a critical aspect of clinical care requiring nuanced decision-making, effective communication, and cultural competence. The findings of our survey underscore significant inter-country variability in resuscitation decision-making practices and the barriers perceived by geriatricians across Europe.

The frequency of resuscitation discussions varied widely, with roughly a quarter (23%) of geriatricians discussing resuscitation in the majority of hospitalised patients, while a fifth (20%) engaged in these conversations with less than 20% of their patients. This variability is consistent with prior studies highlighting disparities in resuscitation decision-making across countries [17, 18]. Notably, Western European geriatricians were more likely to engage in frequent discussions, compared to their counterparts in Northern, Eastern, and Southern Europe. This could reflect differences in healthcare systems, societal attitudes toward end-of-life care, or legal frameworks governing resuscitation orders.

Our results identified a range of barriers to resuscitation discussions, with lack of time being the most frequently cited (50%), followed by cultural or religious beliefs (25%), and discomfort or lack of confidence (21%). The time barrier is particularly notable, as it highlights the competing demands placed on clinicians, potentially limiting opportunities for meaningful discussions, and has been reported by others [19]. Cultural barriers, cited by one in four respondents, emphasise the need for culturally sensitive communication strategies, as differences in attitudes toward death and dying are well-documented across Europe [20–22].

Cultural barriers given by regional cultural differences may also be reflected by our finding, that more respondents in Southern and Eastern European countries thought that “discussing DNR is not well accepted in my country”, compared to colleagues from Western and Northern European countries. The Ethicus Study investigating end-of-life practices in European intensive care units found similar results for Southern and Northern/Western European Countries with differences in distribution of different religions as one explanation [23]. We did not ask about the religion of our respondents, but religious differences across Europe have been shown to affect regional differences on issues such as euthanasia [24]. Alongside religion, Europe is home to a wide expanse of cultures, who vary in their outlook on death, on dying, on old age, and on the role of the healthcare provider, to name but a few. All of these influences can play a role in resuscitation practices.

Confidence in discussing resuscitation was high among respondents, with almost 80% reporting agreement or strong agreement to feeling confident. Female physicians and those with greater experience were more likely to report higher confidence. These findings align with prior research showing that clinical experience and exposure to structured training significantly enhance confidence in sensitive communication tasks [25–27]. However, the survey revealed significant gaps in training, with 41% of respondents reporting no formal education in resuscitation discussions. Those who received training predominantly did so during residency, suggesting that medical schools might be under-emphasising this critical area. Indeed 15% of respondents reported they did not know whether it was legal for physicians to make do-not-resuscitate decisions in cases where a patient lacks capacity. Enhanced training in communication and decision-making around resuscitation has been shown to improve clinician confidence [28]. It is notable that confidence remained high for most respondents, despite this lack of training, highlighting the importance of experience on the ground as training in itself. Considering the wide variety of prevalence of resuscitation discussions, and the reported lack of structured training, there is clearly room for improvement in this area, particularly in Southern and Eastern Europe.

Structured documentation and standardised protocols for DNR orders were also lacking in many countries, with only 55% of respondents reporting the availability of a standardised DNR document. Furthermore, 59% of respondents indicated that preferences for resuscitation were not routinely recorded at hospital admission. These systemic gaps likely contribute to inconsistencies in resuscitation practices and underscore the need for organisational and policy-level interventions across the continent. Others have noted the importance of a standardised policy in this area to facilitate discussion and minimise physician uncertainty [19]. This will be particularly relevant as European populations continue to age, and more and more non-geriatricians will be caring for older adults and those living with frailty in hospital.

Interestingly, 38% of respondents indicated that futile therapies, such as CPR in patients with no perceived chance of survival, were still performed when requested by patients or their next of kin. This finding highlights the tension between respecting patient autonomy and the ethical principle of non-maleficence [28, 29]. It also reflects the potential for misaligned expectations among patients, families, and healthcare providers—a phenomenon reported elsewhere [30]. When patients or their families are keen to have CPR offered in scenarios where healthcare providers believe it will be futile, this suggests a lack of clear communication between both parties. Indeed, a study from the US showed that patients often changed their request for intensive care treatment in case of life threatening events after being told about their realistic outcome [8].

As geriatricians are at the forefront of care for frail older patients, efforts to optimise resuscitation practices are likely to be led by this group. Indeed a Belgian study performed during the Covid-19 pandemic reported that performing comprehensive geriatric assessment (CGA) in patients leads to higher frequency of DNR decision [31]. However, as the population continues to age, more non-geriatricians will be leading care of older adults. Increased use of standardised protocols, and more widespread training for physicians, are likely to be beneficial, alongside the learning that comes with clinical experience, to improve resuscitation decision-making practices across the continent.

## Strengths and limitations

This study has several limitations. The use of an online survey disseminated through professional networks may have introduced selection bias, as respondents who are more engaged with professional organisations may not be representative of all geriatricians. Indeed, we did not get an equal representation across Europe and some regions such as Eastern Europe are under-represented. Additionally, the survey was conducted in English, which may have limited participation from non-English-speaking regions or affected the interpretation of questions, despite our best efforts to minimise this. Our survey did not explore the perspectives of patients and next of kin as our aim was to focus on physicians, however the position of patients and their families should always be taken into consideration when designing strategies for resuscitation decision-making. Finally, the cross-sectional design precludes any causal inferences.

That said, the strengths of this work are represented in the high number of respondents, the use of national societies to disseminate this survey, and the wide-ranging international collaboration between the investigators, who work across seven different European nations.

## Conclusions

Resuscitation decision-making among geriatricians in Europe varies widely, reflecting differences in training, systemic factors, and cultural influences. While cultural heterogeneity will remain across Europe, increased use of standardised protocols, and more widespread training for physicians, are likely to be beneficial, alongside the learning that comes with clinical experience, to improve resuscitation decision-making practices across the continent.

## Supplementary Information

Below is the link to the electronic supplementary material.

Supplementary file1 (DOCX 64 KB)
