# Supplementary material for: Resuscitation discussion practices: a survey of European geriatricians
Source: Eur Geriatr Med. 2025 May 5;16(4):1527–36. doi: 10.1007/s41999-025-01218-8 (PMC12378277; doi:10.1007/s41999-025-01218-8)
Supplement: Supplementary file 1 — Supplementary file1 (DOCX 64 KB) [file 41999_2025_1218_MOESM1_ESM.docx]

**Resuscitation Discussion Practices: A Survey of European Geriatricians**

**SUPPLEMENTARY MATERIAL**

**Supplementary Table 1: Survey Results**

|  | **Question** | **Answers** | **Number (n,%)** |
| --- | --- | --- | --- |
| 1 | With which proportion of admitted patients, independently of morbidity status, do you discuss DNR orders? | 0-20%  20-40%  40-60%  60-80%  80-100%  *Missing* | 96 (20.3)  90 (19.1)  98 (20.8)  79 (16.7)  109 (23.1)  1 (0.2) |
| 2a | I feel confident discussing DNR orders with patients. | Strongly agree  Agree  Neutral  Disagree  Strongly disagree  *Missing* | 161 (34.0)  216 (45.7)  47 (9.9)  42 (8.9)  7 (1.5)  0 |
| 2b | I feel confident discussing DNR orders with patient’s next of kin. | Strongly agree  Agree  Neutral  Disagree  Strongly disagree  *Missing* | 180 (38.1)  234 (49.5)  44 (9.3)  14 (3.0)  1 (0.2)  0 |
| 3 | What do you feel are the barriers or reasons not to discuss resuscitation or talking about death and dying?* | Religious or cultural beliefs  Fear of lawsuits  Inexperience or lack of skills  Embarrassment / feeling uncomfortable  Lack of time  I don’t believe it’s needed  None  Other | 117 (24.7)  44 (9.3)  60 (12.7)  99 (20.9)  236 (49.9)  44 (9.3)  0  17 (15.0) |
| 4 | When do you think discussing DNR orders in the hospital is most appropriate (for those patients without known ceilings of care)? | On admission  A few days after admission  In case of clinical deterioration  Other  *Missing* | 227 (48.0)  117 (24.7)  82 (17.3)  47 (9.9)  0 |
| 5 | With whom do you usually discuss DNR orders for patients with full capacity to make a decision? | Patients  Next of kin  Both patients and next of kin  Neither  *Missing* | 218 (46.1)  42 (8.9)  203 (42.9)  8 (.17)  2 (0.4) |
| 6 | With whom do you usually discuss DNR orders for patients with impaired capacity to make a decision? | Patients  Next of kin / legal representative  Both patients and next of kin  Neither  *Missing* | 19 (4.0)  181 (38.3)  262 (55.4)  7 (1.5)  4 (0.8) |
| 7 | Is it legal in your country to make DNR decisions by medical opinion alone (e.g., the patient has cognitive impairment, lacks capacity, and no legal representative available)? | Yes  No  I don’t know  *Missing* | 341 (72.1)  60 (12.7)  71 (15.0)  1 (0.2) |
| 8 | I feel confident making DNR decisions based on medical opinion alone (e.g. in the example above)? | Strongly agree  Agree  Neutral  Disagree  Strongly disagree  *Missing* | 90 (19.0)  224 (47.4)  73 (15.4)  74 (15.6)  10 (2.1)  2 (0.4) |
| 9 | If there is an advance care plan mentioning a conditional DNR in specific situations, how often do you find it difficult to know whether it should apply in the patient’s current situation? | Always  Often  Rarely  Never  Not applicable  *Missing* | 17 (3.6)  169 (35.7)  210 (44.4)  22 (4.7)  51 (10.8)  4 (0.8) |
| 10 | In a situation where the medical team thinks that a therapy is futile (e.g. resuscitation), how often would this therapy anyway be performed in your country, if the patient or next of kin mandates it? | Always  Often  Rarely  Never  Not applicable  *Missing* | 21 (4.4)  158 (33.4)  264 (55.8)  20 (4.2)  7 (1.5)  3 (0.6) |
| 11 | How often do you re-evaluate DNR orders after making the decision initially (during the same hospitalisation)? | Always  Often  Rarely  Never  *Missing* | 16 (3.4)  145 (30.7)  291 (61.5)  19 (4.0)  2 (0.4) |
| 12 | Is it mandatory in your country to write and assess preferences regarding resuscitation in clinical records on admission? | Yes  No  I don’t know  *Missing* | 132 (27.9)  278 (58.8)  58 (12.3)  5 (1.1) |
| 13 | Does your hospital provide a standardised DNR document? | Yes  No  I don’t know  *Missing* | 260 (55.0)  190 (40.2)  21 (4.4)  2 (0.4) |
| 14 | Discussing DNR orders in my country is well accepted. | Strongly agree  Agree  Neutral  Disagree  Strongly disagree  *Missing* | 85 (18.0)  206 (43.6)  91 (19.2)  74 (15.6)  14 (3.0)  3 (0.6) |
| 15 | Did you receive any training about communication/discussing DNR orders? | In university  In residency / specialty training  Both  No training  *Missing* | 27 (5.7)  170 (35.9)  83 (17.5)  192 (40.6)  1 (0.2) |
| 16 | In your opinion, what needs to change to improve DNR decision making in your country?* | More training  Mandatory discussions  More awareness in society  Nothing  Other | 328 (70.2)  222 (47.0)  387 (82.0)  5 (1.1)  25 (5.3) |
| * = multiple answers possible | | | |

**Supplementary Table 2. Survey results according to individual country (if n≥ 5 respondents in a country)**

| **Country** | **Belgium** | **Denmark** | **Finland** | **France** | **Germany** | **Ireland** | **Italy** | **Netherlands** | **Norway** | **Poland** | **Portugal** | **Spain** | **Switzerland** | **Turkey** | **UK** |
| --- | --- | --- | --- | --- | --- | --- | --- | --- | --- | --- | --- | --- | --- | --- | --- |
| **n** | 11 | 17 | 11 | 13 | 83 | 45 | 56 | 32 | 12 | 7 | 6 | 60 | 20 | 5 | 78 |
| **Baseline data** | | | | | | | | | | | | | | | |
| **Age** | 42.18 (10.36) | 39.35 (5.82) | 47.09 (6.88) | 39.15 (7.30) | 42.06 (9.58) | 41.00 (10.75) | 39.86 (11.36) | 40.81 (9.09) | 45.58 (7.45) | 42.17 (14.03) | 40.83 (6.08) | 42.17 (11.41) | 42.47 (7.76) | 37.00 (7.18) | 42.79 (9.18) |
| **Females (%)** | 8 (72.7) | 12 (70.6) | 7 (63.6) | 7 (53.8) | 47 (56.6) | 26 (57.8) | 34 (61.8) | 30 (93.8) | 5 (41.7) | 4 (57.1) | 5 (83.3) | 46 (79.3) | 9 (45.0) | 3 (60.0) | 48 (61.5) |
| **MD work years** | 16.64 (10.83) | 11.47 (5.58) | 19.55 (7.24) | 11.54 (8.31) | 14.18 (9.07) | 17.38 (10.78) | 13.38 (10.88) | 14.12 (8.12) | 18.25 (6.97) | 15.29 (13.76) | 14.17 (6.01) | 15.15 (10.61) | 13.70 (6.55) | 12.40 (7.89) | 17.94 (9.16) |
| **Geriatric Work years** | 9.91 (7.41) | 7.94 (5.24) | 11.36 (5.94) | 9.46 (4.01) | 6.92 (6.18) | 13.20 (10.38) | 10.89 (10.26) | 10.50 (7.18) | 11.42 (6.35) | 11.43 (9.03) | 6.17 (1.60) | 12.63 (10.46) | 7.40 (5.14) | 5.40 (4.98) | 12.54 (8.39) |
| **With which proportion of admitted patients, independently of morbidity status, do you discuss DNR orders?** | | | | | | | | | | | | | | | |
| **0-20%** | 0 (0.0) | 0 (0.0) | 0 (0.0) | 2 (15.4) | 10 (12.0) | 9 (20.0) | 31 (55.4) | 0 (0.0) | 4 (33.3) | 5 (71.4) | 2 (33.3) | 15 (25.0) | 1 (5.0) | 4 (80.0) | 4 (5.1) |
| **20-40%** | 0 (0.0) | 3 (17.6) | 4 (40.0) | 5 (38.5) | 16 (19.3) | 22 (48.9) | 13 (23.2) | 0 (0.0) | 4 (33.3) | 0 (0.0) | 0 (0.0) | 12 (20.0) | 1 (5.0) | 1 (20.0) | 8 (10.3) |
| **40-60%** | 3 (27.3) | 6 (35.3) | 2 (20.0) | 3 (23.1) | 22 (26.5) | 11 (24.4) | 5 (8.9) | 1 (3.1) | 2 (16.7) | 1 (14.3) | 2 (33.3) | 16 (26.7) | 0 (0.0) | 0 (0.0) | 20 (25.6) |
| **60-80%** | 5 (45.5) | 2 (11.8) | 0 (0.0) | 1 (7.7) | 14 (16.9) | 2 (4.4) | 4 (7.1) | 2 (6.2) | 2 (16.7) | 1 (14.3) | 1 (16.7) | 13 (21.7) | 4 (20.0) | 0 (0.0) | 27 (34.6) |
| **80-100%** | 3 (27.3) | 6 (35.3) | 4 (40.0) | 2 (15.4) | 21 (25.3) | 1 (2.2) | 3 (5.4) | 29 (90.6) | 0 (0.0) | 0 (0.0) | 1 (16.7) | 4 (6.7) | 14 (70.0) | 0 (0.0) | 19 (24.4) |

| **Country** | **Belgium** | **Denmark** | **Finland** | **France** | **Germany** | **Ireland** | **Italy** | **Netherlands** | **Norway** | **Poland** | **Portugal** | **Spain** | **Switzerland** | **Turkey** | **UK** |
| --- | --- | --- | --- | --- | --- | --- | --- | --- | --- | --- | --- | --- | --- | --- | --- |
| **n** | 11 | 17 | 11 | 13 | 83 | 45 | 56 | 32 | 12 | 7 | 6 | 60 | 20 | 5 | 78 |
| **I feel confident discussing DNR orders with patients** | | | | | | | | | | | | | | | |
| **Agree** | 8 (72.7) | 9 (52.9) | 5 (45.5) | 7 (53.8) | 47 (56.6) | 24 (53.3) | 20 (35.7) | 13 (40.6) | 5 (41.7) | 1 (14.3) | 3 (50.0) | 29 (48.3) | 7 (35.0) | 1 (20.0) | 29 (37.2) |
| **Disagree** | 1 (9.1) | 0 (0.0) | 0 (0.0) | 1 (7.7) | 2 (2.4) | 1 (2.2) | 18 (32.1) | 1 (3.1) | 1 (8.3) | 3 (42.9) | 2 (33.3) | 8 (13.3) | 0 (0.0) | 1 (20.0) | 0 (0.0) |
| **Neutral** | 1 (9.1) | 0 (0.0) | 0 (0.0) | 1 (7.7) | 6 (7.2) | 1 (2.2) | 10 (17.9) | 0 (0.0) | 3 (25.0) | 1 (14.3) | 1 (16.7) | 14 (23.3) | 0 (0.0) | 2 (40.0) | 2 (2.6) |
| **Strongly agree** | 1 (9.1) | 8 (47.1) | 6 (54.5) | 4 (30.8) | 28 (33.7) | 19 (42.2) | 5 (8.9) | 18 (56.2) | 3 (25.0) | 0 (0.0) | 0 (0.0) | 8 (13.3) | 13 (65.0) | 0 (0.0) | 47 (60.3) |
| **Strongly disagree** | 0 (0.0) | 0 (0.0) | 0 (0.0) | 0 (0.0) | 0 (0.0) | 0 (0.0) | 3 (5.4) | 0 (0.0) | 0 (0.0) | 2 (28.6) | 0 (0.0) | 1 (1.7) | 0 (0.0) | 1 (20.0) | 0 (0.0) |
| **I feel confident discussing DNR orders with patient’s next of kin.** | | | | | | | | | | | | | | | |
| **Agree** | 10 (90.9) | 10 (58.8) | 6 (54.5) | 7 (53.8) | 47 (56.6) | 18 (40.0) | 25 (44.6) | 15 (46.9) | 7 (58.3) | 5 (71.4) | 4 (66.7) | 28 (46.7) | 10 (50.0) | 1 (20.0) | 30 (38.5) |
| **Disagree** | 0 (0.0) | 0 (0.0) | 0 (0.0) | 0 (0.0) | 1 (1.2) | 0 (0.0) | 6 (10.7) | 1 (3.1) | 0 (0.0) | 1 (14.3) | 0 (0.0) | 2 (3.3) | 0 (0.0) | 2 (40.0) | 0 (0.0) |
| **Neutral** | 0 (0.0) | 0 (0.0) | 1 (9.1) | 0 (0.0) | 12 (14.5) | 3 (6.7) | 9 (16.1) | 1 (3.1) | 2 (16.7) | 0 (0.0) | 1 (16.7) | 9 (15.0) | 0 (0.0) | 1 (20.0) | 2 (2.6) |
| **Strongly agree** | 1 (9.1) | 7 (41.2) | 4 (36.4) | 6 (46.2) | 23 (27.7) | 24 (53.3) | 16 (28.6) | 15 (46.9) | 3 (25.0) | 0 (0.0) | 1 (16.7) | 21 (35.0) | 10 (50.0) | 1 (20.0) | 46 (59.0) |
| **Strongly disagree** | 0 (0.0) | 0 (0.0) | 0 (0.0) | 0 (0.0) | 0 (0.0) | 0 (0.0) | 0 (0.0) | 0 (0.0) | 0 (0.0) | 1 (14.3) | 0 (0.0) | 0 (0.0) | 0 (0.0) | 0 (0.0) | 0 (0.0) |

| **Country** | **Belgium** | **Denmark** | **Finland** | **France** | **Germany** | **Ireland** | **Italy** | **Netherlands** | **Norway** | **Poland** | **Portugal** | **Spain** | **Switzerland** | **Turkey** | **UK** |
| --- | --- | --- | --- | --- | --- | --- | --- | --- | --- | --- | --- | --- | --- | --- | --- |
| **n** | **11** | **17** | **11** | **13** | **83** | **45** | **56** | **32** | **12** | **7** | **6** | **60** | **20** | **5** | **78** |
| **What do you feel are the barriers or reasons not to discuss resuscitation or talking about death and dying?** | | | | | | | | | | | | | | | |
| **Religious cultural** | 6 (54.5) | 3 (17.6) | 3 (27.3) | 0 (0.0) | 16 (19.3) | 3 (6.7) | 14 (25.0) | 18 (56.2) | 2 (16.7) | 3 (42.9) | 4 (66.7) | 11 (18.3) | 5 (25.0) | 4 (80.0) | 20 (25.6) |
| **Fear of law** | 0 (0.0) | 0 (0.0) | 0 (0.0) | 0 (0.0) | 8 (9.6) | 0 (0.0) | 16 (28.6) | 0 (0.0) | 0 (0.0) | 4 (57.1) | 0 (0.0) | 5 (8.3) | 3 (15.0) | 5 (100.0) | 1 (1.3) |
| **Inexperience** | 1 (9.1) | 0 (0.0) | 1 (9.1) | 1 (7.7) | 6 (7.2) | 2 (4.4) | 22 (39.3) | 0 (0.0) | 0 (0.0) | 2 (28.6) | 1 (16.7) | 18 (30.0) | 2 (10.0) | 0 (0.0) | 0 (0.0) |
| **Embarrassment** | 2 (18.2) | 3 (17.6) | 0 (0.0) | 2 (15.4) | 18 (21.7) | 8 (17.8) | 17 (30.4) | 2 (6.2) | 4 (33.3) | 3 (42.9) | 3 (50.0) | 22 (36.7) | 3 (15.0) | 0 (0.0) | 6 (7.7) |
| **Lack of time** | 5 (45.5) | 14 (82.4) | 6 (54.5) | 9 (69.2) | 38 (45.8) | 26 (57.8) | 8 (14.3) | 12 (37.5) | 9 (75.0) | 2 (28.6) | 3 (50.0) | 25 (41.7) | 12 (60.0) | 1 (20.0) | 58 (74.4) |
| **I don’t believe it’s needed** | 1 (9.1) | 3 (17.6) | 1 (9.1) | 1 (7.7) | 11 (13.3) | 7 (15.6) | 1 (1.8) | 1 (3.1) | 2 (16.7) | 1 (14.3) | 0 (0.0) | 2 (3.3) | 1 (5.0) | 0 (0.0) | 10 (12.8) |
| **Other** | 2 (18.2) | 3 (17.6) | 1 (9.1) | 2 (15.4) | 8 (9.6) | 10 (22.2) | 4 (7.1) | 4 (12.5) | 5 (41.7) | 2 (28.6) | 0 (0.0) | 6 (10.0) | 2 (10.0) | 0 (0.0) | 18 (23.1) |

| **Country** | **Belgium** | **Denmark** | **Finland** | **France** | **Germany** | **Ireland** | **Italy** | **Netherlands** | **Norway** | **Poland** | **Portugal** | **Spain** | **Switzerland** | **Turkey** | **UK** |
| --- | --- | --- | --- | --- | --- | --- | --- | --- | --- | --- | --- | --- | --- | --- | --- |
| **n** | **11** | **17** | **11** | **13** | **83** | **45** | **56** | **32** | **12** | **7** | **6** | **60** | **20** | **5** | **78** |
| **With whom do you usually discuss DNR orders for patients with full capacity to make a decision?** | | | | | | | | | | | | | | | |
| **Both patients and next of kin (patients’ family)** | | | | | | | | | | | | | | | |
|  | 3 (27.3) | 7 (41.2) | 3 (27.3) | 7 (53.8) | 23 (27.7) | 24 (53.3) | 27 (48.2) | 24 (75.0) | 4 (33.3) | 1 (14.3) | 2 (33.3) | 34 (56.7) | 1 (5.0) | 1 (20.0) | 35 (44.9) |
| **Neither** | | | | | | | | | | | | | | | |
|  | 0 (0.0) | 0 (0.0) | 0 (0.0) | 0 (0.0) | 0 (0.0) | 0 (0.0) | 1 (1.8) | 0 (0.0) | 1 (8.3) | 1 (14.3) | 1 (16.7) | 0 (0.0) | 0 (0.0) | 2 (40.0) | 0 (0.0) |
| **Next of kin (family)** | | | | | | | | | | | | | | | |
|  | 0 (0.0) | 0 (0.0) | 0 (0.0) | 0 (0.0) | 0 (0.0) | 0 (0.0) | 17 (30.4) | 0 (0.0) | 0 (0.0) | 5 (71.4) | 2 (33.3) | 12 (20.0) | 0 (0.0) | 1 (20.0) | 0 (0.0) |
| **Patients** | | | | | | | | | | | | | | | |
|  | 8 (72.7) | 10 (58.8) | 8 (72.7) | 5 (38.5) | 59 (71.1) | 21 (46.7) | 11 (19.6) | 8 (25.0) | 7 (58.3) | 0 (0.0) | 1 (16.7) | 13 (21.7) | 19 (95.0) | 1 (20.0) | 42 (53.8) |
| **With whom do you usually discuss DNR orders for patients with impaired capacity to make a decision?** | | | | | | | | | | | | | | | |
| **Both patients and next of kin (family) / legal representative** | | | | | | | | | | | | | | | |
|  | 8 (72.7) | 12 (70.6) | 10 (90.9) | 8 (61.5) | 60 (72.3) | 25 (55.6) | 12 (21.4) | 27 (84.4) | 7 (58.3) | 1 (14.3) | 2 (33.3) | 20 (33.3) | 16 (80.0) | 1 (20.0) | 47 (60.3) |
| **Neither** | | | | | | | | | | | | | | | |
|  | 0 (0.0) | 0 (0.0) | 0 (0.0) | 0 (0.0) | 0 (0.0) | 0 (0.0) | 0 (0.0) | 0 (0.0) | 2 (16.7) | 1 (14.3) | 0 (0.0) | 0 (0.0) | 0 (0.0) | 2 (40.0) | 0 (0.0) |
| **Next of kin (patients’ family) /legal representative** | | | | | | | | | | | | | | | |
|  | 3 (27.3) | 5 (29.4) | 1 (9.1) | 5 (38.5) | 19 (22.9) | 15 (33.3) | 42 (75.0) | 4 (12.5) | 1 (8.3) | 4 (57.1) | 4 (66.7) | 36 (60.0) | 1 (5.0) | 2 (40.0) | 30 (38.5) |
| **Patients where possible** | | | | | | | | | | | | | | | |
|  | 0 (0.0) | 0 (0.0) | 0 (0.0) | 0 (0.0) | 3 (3.6) | 5 (11.1) | 1 (1.8) | 1 (3.1) | 2 (16.7) | 1 (14.3) | 0 (0.0) | 2 (3.3) | 3 (15.0) | 0 (0.0) | 1 (1.3) |

| **Country** | **Belgium** | **Denmark** | **Finland** | **France** | **Germany** | **Ireland** | **Italy** | **Netherlands** | **Norway** | **Poland** | **Portugal** | **Spain** | **Switzerland** | **Turkey** | **UK** |
| --- | --- | --- | --- | --- | --- | --- | --- | --- | --- | --- | --- | --- | --- | --- | --- |
| **n** | **11** | **17** | **11** | **13** | **83** | **45** | **56** | **32** | **12** | **7** | **6** | **60** | **20** | **5** | **78** |
| **Is it legal in your country to make DNR decisions by medical opinion alone (e.g., patient has cognitive impairment, lacks capacity, and no legal representative available)?** | | | | | | | | | | | | | | | |
| **I don´t know** | 4 (36.4) | 0 (0.0) | 0 (0.0) | 0 (0.0) | 20 (24.1) | 4 (8.9) | 20 (35.7) | 0 (0.0) | 0 (0.0) | 0 (0.0) | 1 (16.7) | 17 (28.3) | 0 (0.0) | 0 (0.0) | 2 (2.6) |
| **No** | 0 (0.0) | 0 (0.0) | 0 (0.0) | 7 (53.8) | 8 (9.6) | 0 (0.0) | 12 (21.4) | 0 (0.0) | 0 (0.0) | 2 (28.6) | 1 (16.7) | 7 (11.7) | 0 (0.0) | 5 (100.0) | 9 (11.5) |
| **Yes** | 7 (63.6) | 17 (100) | 11 (100) | 6 (46.2) | 55 (66.3) | 41 (91.1) | 24 (42.9) | 32 (100) | 12 (100) | 5 (71.4) | 4 (66.7) | 35 (58.3) | 20 (100) | 0 (0.0) | 67 (85.9) |
| **I feel confident making DNR decisions based on medical opinion alone (e.g. in the example above)?** | | | | | | | | | | | | | | | |
|  | 0 (0.0) | 0 (0.0) | 0 (0.0) | 0 (0.0) | 0 (0.0) | 0 (0.0) | 0 (0.0) | 0 (0.0) | 0 (0.0) | 0 (0.0) | 0 (0.0) | 1 (1.7) | 0 (0.0) | 0 (0.0) | 1 (1.3) |
| **Agree** | 6 (54.5) | 9 (52.9) | 6 (54.5) | 4 (30.8) | 35 (42.2) | 26 (57.8) | 22 (39.3) | 21 (65.6) | 10 (83.3) | 4 (57.1) | 4 (66.7) | 31 (51.7) | 7 (35.0) | 1 (20.0) | 32 (41.0) |
| **Disagree** | 1 (9.1) | 0 (0.0) | 0 (0.0) | 4 (30.8) | 23 (27.7) | 1 (2.2) | 15 (26.8) | 1 (3.1) | 0 (0.0) | 1 (14.3) | 1 (16.7) | 14 (23.3) | 4 (20.0) | 1 (20.0) | 3 (3.8) |
| **Neutral** | 2 (18.2) | 3 (17.6) | 0 (0.0) | 3 (23.1) | 13 (15.7) | 5 (11.1) | 15 (26.8) | 5 (15.6) | 0 (0.0) | 1 (14.3) | 1 (16.7) | 9 (15.0) | 2 (10.0) | 1 (20.0) | 8 (10.3) |
| **Strongly agree** | 2 (18.2) | 5 (29.4) | 5 (45.5) | 2 (15.4) | 9 (10.8) | 13 (28.9) | 3 (5.4) | 5 (15.6) | 2 (16.7) | 1 (14.3) | 0 (0.0) | 2 (3.3) | 7 (35.0) | 1 (20.0) | 32 (41.0) |
| **Strongly disagree** | 0 (0.0) | 0 (0.0) | 0 (0.0) | 0 (0.0) | 3 (3.6) | 0 (0.0) | 1 (1.8) | 0 (0.0) | 0 (0.0) | 0 (0.0) | 0 (0.0) | 3 (5.0) | 0 (0.0) | 1 (20.0) | 2 (2.6) |
| **If there is an advance care plan mentioning a conditional DNR in specific situations, how often do you find it difficult to know whether it should apply in the patient’s current situation?** | | | | | | | | | | | | | | | |
| **Always** | 0 (0.0) | 0 (0.0) | 0 (0.0) | 2 (15.4) | 6 (7.2) | 0 (0.0) | 4 (7.1) | 0 (0.0) | 0 (0.0) | 0 (0.0) | 0 (0.0) | 0 (0.0) | 1 (5.0) | 0 (0.0) | 2 (2.6) |
| **Often** | 2 (18.2) | 6 (35.3) | 3 (27.3) | 4 (30.8) | 52 (62.7) | 3 (6.7) | 27 (48.2) | 7 (21.9) | 3 (25.0) | 1 (14.3) | 3 (50.0) | 27 (45.0) | 9 (45.0) | 1 (20.0) | 13 (16.7) |
| **Rarely** | 8 (72.7) | 9 (52.9) | 4 (36.4) | 5 (38.5) | 22 (26.5) | 29 (64.4) | 17 (30.4) | 20 (62.5) | 6 (50.0) | 4 (57.1) | 3 (50.0) | 30 (50.0) | 9 (45.0) | 2 (40.0) | 38 (48.7) |
| **Never** | 0 (0.0) | 0 (0.0) | 3 (27.3) | 1 (7.7) | 0 (0.0) | 2 (4.4) | 3 (5.4) | 1 (3.1) | 1 (8.3) | 0 (0.0) | 0 (0.0) | 0 (0.0) | 1 (5.0) | 1 (20.0) | 7 (9.0) |
| **Not applicable** | 0 (0.0) | 2 (11.8) | 1 (9.1) | 0 (0.0) | 2 (2.4) | 11 (24.4) | 5 (8.9) | 4 (12.5) | 2 (16.7) | 2 (28.6) | 0 (0.0) | 2 (3.3) | 0 (0.0) | 1 (20.0) | 18 (23.1) |

| **Country** | **Belgium** | **Denmark** | **Finland** | **France** | **Germany** | **Ireland** | **Italy** | **Netherlands** | **Norway** | **Poland** | **Portugal** | **Spain** | **Switzerland** | **Turkey** | **UK** |
| --- | --- | --- | --- | --- | --- | --- | --- | --- | --- | --- | --- | --- | --- | --- | --- |
| **n** | **11** | **17** | **11** | **13** | **83** | **45** | **56** | **32** | **12** | **7** | **6** | **60** | **20** | **5** | **78** |
| **In a situation where the medical team thinks that a therapy is futile (e.g. resuscitation), how often would this therapy anyway be performed in your country, if the patient or next of kin mandates it?** | | | | | | | | | | | | | | | |
| **Always** | 0 (0.0) | 0 (0.0) | 0 (0.0) | 0 (0.0) | 5 (6.0) | 0 (0.0) | 4 (7.1) | 1 (3.1) | 0 (0.0) | 0 (0.0) | 0 (0.0) | 4 (6.7) | 0 (0.0) | 2 (40.0) | 1 (1.3) |
| **Never** | 1 (9.1) | 2 (11.8) | 1 (9.1) | 2 (15.4) | 1 (1.2) | 2 (4.4) | 1 (1.8) | 2 (6.2) | 1 (8.3) | 0 (0.0) | 0 (0.0) | 1 (1.7) | 0 (0.0) | 0 (0.0) | 4 (5.1) |
| **Not applicable** | 1 (9.1) | 0 (0.0) | 0 (0.0) | 1 (7.7) | 3 (3.6) | 0 (0.0) | 0 (0.0) | 0 (0.0) | 0 (0.0) | 0 (0.0) | 0 (0.0) | 0 (0.0) | 0 (0.0) | 1 (20.0) | 1 (1.3) |
| **Often** | 2 (18.2) | 4 (23.5) | 0 (0.0) | 1 (7.7) | 42 (50.6) | 15 (33.3) | 29 (51.8) | 6 (18.8) | 1 (8.3) | 4 (57.1) | 3 (50.0) | 22 (36.7) | 6 (30.0) | 2 (40.0) | 16 (20.5) |
| **Rarely** | 7 (63.6) | 11 (64.7) | 10 (90.9) | 9 (69.2) | 30 (36.1) | 28 (62.2) | 22 (39.3) | 23 (71.9) | 10 (83.3) | 3 (42.9) | 3 (50.0) | 32 (53.3) | 14 (70.0) | 0 (0.0) | 56 (71.8) |
| **How often do you re-evaluate DNR orders after making the decision initially (during the same hospitalisation)?** | | | | | | | | | | | | | | | |
| **Missing** | 0 (0.0) | 0 (0.0) | 1 (9.1) | 0 (0.0) | 0 (0.0) | 0 (0.0) | 0 (0.0) | 0 (0.0) | 0 (0.0) | 0 (0.0) | 0 (0.0) | 1 (1.7) | 0 (0.0) | 0 (0.0) | 0 (0.0) |
| **Always** | 0 (0.0) | 0 (0.0) | 1 (9.1) | 2 (15.4) | 1 (1.2) | 2 (4.4) | 1 (1.8) | 0 (0.0) | 0 (0.0) | 1 (14.3) | 0 (0.0) | 4 (6.7) | 0 (0.0) | 1 (20.0) | 3 (3.8) |
| **Never** | 0 (0.0) | 0 (0.0) | 2 (18.2) | 0 (0.0) | 1 (1.2) | 5 (11.1) | 3 (5.4) | 0 (0.0) | 2 (16.7) | 1 (14.3) | 0 (0.0) | 1 (1.7) | 0 (0.0) | 1 (20.0) | 0 (0.0) |
| **Often** | 8 (72.7) | 4 (23.5) | 0 (0.0) | 4 (30.8) | 15 (18.1) | 16 (35.6) | 18 (32.1) | 8 (25.0) | 4 (33.3) | 3 (42.9) | 2 (33.3) | 29 (48.3) | 6 (30.0) | 2 (40.0) | 21 (26.9) |
| **Rarely** | 3 (27.3) | 13 (76.5) | 7 (63.6) | 7 (53.8) | 66 (79.5) | 22 (48.9) | 34 (60.7) | 24 (75.0) | 6 (50.0) | 2 (28.6) | 4 (66.7) | 25 (41.7) | 14 (70.0) | 1 (20.0) | 54 (69.2) |
| **Is it mandatory in your country to write and assess preferences regarding resuscitation in clinical records on admission?** | | | | | | | | | | | | | | | |
| **I don´t**  **know** | 2 (18.2) | 1 (5.9) | 0 (0.0) | 4 (30.8) | 16 (19.3) | 2 (4.4) | 9 (16.1) | 2 (6.2) | 0 (0.0) | 0 (0.0) | 1 (16.7) | 9 (15.0) | 2 (10.0) | 0 (0.0) | 6 (7.7) |
| **No** | 4 (36.4) | 8 (47.1) | 8 (72.7) | 5 (38.5) | 52 (62.7) | 39 (86.7) | 40 (71.4) | 6 (18.8) | 10 (83.3) | 6 (85.7) | 3 (50.0) | 32 (53.3) | 5 (25.0) | 5 (100.0) | 45 (57.7) |
| **Yes** | 5 (45.5) | 8 (47.1) | 3 (27.3) | 4 (30.8) | 15 (18.1) | 4 (8.9) | 6 (10.7) | 24 (75.0) | 1 (8.3) | 0 (0.0) | 1 (16.7) | 18 (30.0) | 13 (65.0) | 0 (0.0) | 27 (34.6) |

| **Country** | **Belgium** | **Denmark** | **Finland** | **France** | **Germany** | **Ireland** | **Italy** | **Netherlands** | **Norway** | **Poland** | **Portugal** | **Spain** | **Switzerland** | **Turkey** | **UK** |
| --- | --- | --- | --- | --- | --- | --- | --- | --- | --- | --- | --- | --- | --- | --- | --- |
| **n** | **11** | **17** | **11** | **13** | **83** | **45** | **56** | **32** | **12** | **7** | **6** | **60** | **20** | **5** | **78** |
| **Does your hospital provide a standardised DNR document?** | | | | | | | | | | | | | | | |
| **I don’t**  **know** | 0 (0.0) | 0 (0.0) | 0 (0.0) | 1 (7.7) | 4 (4.8) | 1 (2.2) | 6 (10.7) | 1 (3.1) | 1 (8.3) | 0 (0.0) | 1 (16.7) | 4 (6.7) | 1 (5.0) | 0 (0.0) | 0 (0.0) |
| **No** | 0 (0.0) | 5 (29.4) | 5 (45.5) | 7 (53.8) | 28 (33.7) | 4 (8.9) | 49 (87.5) | 4 (12.5) | 10 (83.3) | 6 (85.7) | 4 (66.7) | 47 (78.3) | 5 (25.0) | 5 (100.0) | 0 (0.0) |
| **Yes** | 11 (100) | 12 (70.6) | 6 (54.5) | 5 (38.5) | 51 (61.4) | 39 (86.7) | 1 (1.8) | 27 (84.4) | 1 (8.3) | 1 (14.3) | 1 (16.7) | 8 (13.3) | 14 (70.0) | 0 (0.0) | 78 (100) |
| **Discussing DNR orders in my country is well accepted.** | | | | | | | | | | | | | | | |
| **Strongly agree** | **0 (0.0)** | 7 (41.2) | 3 (27.3) | 2 (15.4) | 10 (12.0) | 7 (15.6) | 1 (1.8) | 12 (37.5) | 4 (33.3) | 0 (0.0) | 1 (16.7) | 5 (8.3) | 9 (45.0) | 0 (0.0) | 24 (30.8) |
| **Agree** | 9 (81.8) | 9 (52.9) | 7 (63.6) | 7 (53.8) | 43 (51.8) | 26 (57.8) | 13 (23.2) | 19 (59.4) | 5 (41.7) | 1 (14.3) | 2 (33.3) | 18 (30.0) | 10 (50.0) | 0 (0.0) | 37 (47.4) |
| **Neutral** | 2 (18.2) | 1 (5.9) | 0 (0.0) | 3 (23.1) | 20 (24.1) | 8 (17.8) | 10 (17.9) | 1 (3.1) | 2 (16.7) | 0 (0.0) | 1 (16.7) | 18 (30.0) | 1 (5.0) | 0 (0.0) | 14 (17.9) |
| **Disagree** | 0 (0.0) | 0 (0.0) | 1 (9.1) | 1 (7.7) | 9 (10.8) | 4 (8.9) | 26 (46.4) | 0 (0.0) | 1 (8.3) | 3 (42.9) | 1 (16.7) | 17 (28.3) | 0 (0.0) | 3 (60.0) | 3 (3.8) |
| **Strongly disagree** | 0 (0.0) | 0 (0.0) | 0 (0.0) | 0 (0.0) | 0 (0.0) | 0 (0.0) | 6 (10.7) | 0 (0.0) | 0 (0.0) | 3 (42.9) | 1 (16.7) | 0 (0.0) | 0 (0.0) | 2 (40.0) | 0 (0.0) |
| **Did you receive any training about communication/ discussing DNR orders?** | | | | | | | | | | | | | | | |
| **Both in university and residency** | 1 (9.1) | 2 (11.8) | 1 (9.1) | 2 (15.4) | 2 (2.4) | 7 (15.6) | 3 (5.4) | 8 (25.0) | 5 (41.7) | 1 (14.3) | 0 (0.0) | 9 (15.0) | 7 (35.0) | 1 (20.0) | 33 (42.3) |
| **Residency** | 3 (27.3) | 7 (41.2) | 7 (63.6) | 7 (53.8) | 12 (14.5) | 21 (46.7) | 7 (12.5) | 17 (53.1) | 5 (41.7) | 3 (42.9) | 2 (33.3) | 26 (43.3) | 10 (50.0) | 0 (0.0) | 37 (47.4) |
| **Universi-ty** | 3 (27.3) | 3 (17.6) | 0 (0.0) | 0 (0.0) | 7 (8.4) | 2 (4.4) | 4 (7.1) | 1 (3.1) | 0 (0.0) | 2 (28.6) | 0 (0.0) | 2 (3.3) | 0 (0.0) | 0 (0.0) | 2 (2.6) |
| **No training** | 4 (36.4) | 5 (29.4) | 3 (27.3) | 4 (30.8) | 62 (74.7) | 15 (33.3) | 42 (75.0) | 6 (18.8) | 2 (16.7) | 1 (14.3) | 4 (66.7) | 22 (36.7) | 3 (15.0) | 4 (80.0) | 6 (7.7) |

**Supplementary Table 3. Factors associated with frequency of discussing resuscitation (changing reference category of European area)**

|  | **Discussing Resuscitation with ≥60% patients** | | | | | | | | |
| --- | --- | --- | --- | --- | --- | --- | --- | --- | --- |
|  | *Odds Ratio* | *95% CI* | *p-value* | *Odds Ratio* | *95% CI* | *p-value* | *Odds Ratio* | *95% CI* | *p-value* |
| Age (years) | 0.99 | 0.96 – 1.03 | 0.650 | 0.99 | 0.96 – 1.03 | 0.650 | 0.99 | 0.96 – 1.03 | 0.650 |
| Female sex (vs male) | 1.67 | 1.10 – 2.56 | 0.018 | 1.67 | 1.10 – 2.56 | 0.018 | 1.67 | 1.10 – 2.56 | 0.018 |
| Experience in geriatric field (years) | | | | | | | | | |
| <10 | Ref. | | | Ref. | | | Ref. | | |
| 10-19 | 1.32 | 0.76 – 2.30 | 0.323 | 1.32 | 0.76 – 2.30 | 0.323 | 1.32 | 0.76 – 2.30 | 0.323 |
| ≥20 | 1.03 | 0.44 – 2.43 | 0.942 | 1.03 | 0.44 – 2.43 | 0.942 | 1.03 | 0.44 – 2.43 | 0.942 |
| European region | | | | | | | | | |
| Northern | Ref. | | | 2.14 | 0.55 – 10.67 | 0.301 | 1.94 | 0.86 – 4.28 | 0.103 |
| Western | 2.32 | 1.18 – 4.76 | 0.018 | 4.96 | 1.51 – 22.35 | 0.016 | 4.50 | 2.75 – 7.55 | <0.001 |
| Eastern | 0.47 | 0.09 – 1.81 | 0.301 | Ref. | | | 0.91 | 0.19 – 3.15 | 0.887 |
| Southern | 0.52 | 0.23 – 1.16 | 0.103 | 1.10 | 0.32 – 5.13 | 0.887 | Ref. | | |

CI: Confidence Interval

**Supplementary Table 4. Factors associated with confidence in discussing resuscitation (changing reference category of European area)**

|  | **Confidence in discussing resuscitation** | | | | | | | | |
| --- | --- | --- | --- | --- | --- | --- | --- | --- | --- |
|  | *Odds Ratio* | *95% CI* | *p-value* | *Odds Ratio* | *95% CI* | *p-value* | *Odds Ratio* | *95% CI* | *p-value* |
| Age (years) | 1.01 | 0.97 – 1.06 | 0.603 | 1.01 | 0.97 – 1.06 | 0.603 | 1.01 | 0.97 – 1.06 | 0.603 |
| Female sex (vs male) | 2.03 | 1.10 – 3.81 | 0.024 | 2.03 | 1.10 – 3.81 | 0.024 | 2.03 | 1.10 – 3.81 | 0.024 |
| Experience in geriatric field (years) | | | | | | | | | |
| <10 | Ref. | | | Ref. | | | Ref. | | |
| 10-19 | 0.85 | 0.37 – 1.94 | 0.695 | 0.85 | 0.37 – 1.94 | 0.695 | 0.85 | 0.37 – 1.94 | 0.695 |
| ≥20 | 7.53 | 1.93 – 32.35 | 0.005 | 7.53 | 1.93 – 32.35 | 0.005 | 7.53 | 1.93 – 32.3 | 0.005 |
| European region |  |  |  |  |  |  |  |  |  |
| Northern | Ref. | | | 32.99 | 7.16 – 185.3 | <0.001 | 10.94 | 4.09 – 35.2 | <0.001 |
| Western | 1.82 | 0.56 – 5.03 | 0.272 | 60.16 | 16.22 – 265.81 | <0.001 | 19.95 | 10.59 – 39.6 | <0.001 |
| Eastern | 0.03 | 0.01 – 0.14 | <0.001 | Ref. | | | 0.33 | 0.08 – 1.15 | 0.097 |
| Southern | 0.09 | 0.03 – 0.24 | <0.001 | 3.02 | 0.87 – 12.25 | 0.097 | Ref. | | |

CI: Confidence Interval
